# A Direct Role for the CD1b Endogenous Spacer in the Recognition of a *Mycobacterium tuberculosis* Antigen by T-Cell Receptors

**DOI:** 10.3389/fimmu.2020.566710

**Published:** 2020-10-14

**Authors:** Frank Camacho, Ernesto Moreno, Luis F. Garcia-Alles, Glay Chinea Santiago, Martine Gilleron, Aleikar Vasquez, Yee Siew Choong, Fátima Reyes, Mohd Nor Norazmi, Maria E. Sarmiento, Armando Acosta

**Affiliations:** ^1^Biologicals Sciences School, University of Concepcion, Concepcion, Chile; ^2^Faculty of Basic Sciences, University of Medellin, Medellin, Colombia; ^3^TBI, Université de Toulouse, CNRS, INRA, INSA, Toulouse, France; ^4^Center for Genetic Engineering and Biotechnology, Havana, Cuba; ^5^Institut de Pharmacologie et Biologie Structurale, Université de Toulouse, Toulouse, France; ^6^Institute for Research in Molecular Medicine (INFORMM), Universiti Sains Malaysia, Minden, Malaysia; ^7^School of Health Sciences, Health Campus, Universiti Sains Malaysia, Kubang Kerian, Malaysia

**Keywords:** CD1b, *Mycobacterium tuberculosis*, sulfoglycolipids, Ac_2_SGL, scTCR, endogenous spacer

## Abstract

Lipids, glycolipids and lipopeptides derived from *Mycobacterium tuberculosis* (Mtb) are presented to T cells by monomorphic molecules known as CD1. This is the case of the Mtb-specific sulfoglycolipid Ac_2_SGL, which is presented by CD1b molecules and is recognized by T cells found in tuberculosis (TB) patients and in individuals with latent infections. Our group, using filamentous phage display technology, obtained two specific ligands against the CD1b-Ac_2_SGL complex: (i) a single chain T cell receptor (scTCR) from a human T cell clone recognizing the CD1b-AcSGL complex; and (ii) a light chain domain antibody (dAbκ11). Both ligands showed lower reactivity to a synthetic analog of Ac_2_SGL (SGL12), having a shorter acyl chain as compared to the natural antigen. Here we put forward the hypothesis that the CD1b endogenous spacer lipid (EnSpacer) plays an important role in the recognition of the CD1b-Ac_2_SGL complex by specific T cells. To support this hypothesis we combined: (a) molecular binding assays for both the scTCR and the dAbκ11 antibody domain against a small panel of synthetic Ac_2_SGL analogs having different acyl chains, (b) molecular modeling of the CD1b-Ac_2_SGL/EnSpacer complex, and (c) modeling of the interactions of this complex with the scTCR. Our results contribute to understand the mechanisms of lipid presentation by CD1b molecules and their interactions with T-cell receptors and other specific ligands, which may help to develop specific tools targeting Mtb infected cells for therapeutic and diagnostic applications.

## Introduction

Tuberculosis (TB) is a contagious disease mainly caused by *Mycobacterium tuberculosis* (Mtb) (1), being the leading cause of mortality due to infectious agents (1). The low sensitivity of diagnostic methods, the insufficient therapeutic coverage, the emergence of strains that are resistant to therapy, and the lack of an effective vaccine demand the development of new diagnostic and therapeutic approaches (2, 3).

Human leukocyte antigens (HLA), which are highly polymorphic (more than 26,000 alleles) (4, 5), present a variety of Mtb peptides to T-cells, which is considered a key element in the immune response against Mtb (6–8). On the other hand, lipids, glycolipids, and lipopeptides, are presented to T cells by the non-polymorphic CD1 molecules (9). Several Mtb lipid antigens are presented by CD1 molecules such as CD1a, CD1b, CD1c, and CD1d, increasing the breadth of the T-cell responses to Mtb (10–12).

A variety of Mtb lipid antigens are presented to T cells in association with CD1b (10–12). One mycobacterial antigen, in particular, belonging to the group of diacylated sulfoglycolipids and identified as 2-palmitoyl or 2-stearoyl-3-hydroxyphthioceranoyl-2′-sulfate-α-α’-d-trehalose (Ac_2_SGL), was found to be expressed by virulent Mtb bacilli and presented by CD1b (13). This CD1b-Ac_2_SGL complex was able to stimulate specific T cells found in TB patients and latently infected individuals, but not in healthy-tuberculin skin test (TST) negative individuals (13).

The process of identifying relevant Mtb antigens from purified lipid fractions carried out by Gilleron et al. (2004) (13) was based on the use of several CD1-restricted T cell clones from a healthy donor highly reactive to TST (13). One of these clones, named Z4B27, was specific for Ac_2_SLG, thus allowing its identification as a novel Mtb antigen. The specificity of this clone, as well as the structural requirements conferring antigenicity to Ac_2_SLG, were further studied using a panel of 17 synthetic analogs sharing the same sulfated trehalose head but differing in their lipid tails (14). Replacement of the multi-methyl-branched fatty acid by conventional fatty acids at the 3-position of the trehalose sulfate retained binding to CD1b but prevented T cell stimulation. Among the 17 synthetic sulfoglycolipids that were tested, the analog coded as SGL12 was one of the two most active, although with a lower potency as compared to the native antigen. The observed differences in T-cell stimulating capabilities were attributed to differences in length, methyl branches and stereochemistry between the acyl chains of these compounds (14).

CD1 molecules have a deep and hydrophobic antigen-binding groove that allow the presentation of large hydrophobic antigens (15). Among the members of the human CD1 family, CD1b shows the largest binding groove, capable of accommodating hydrophobic chains of about 70 carbons (16, 17). After biosynthesis, the integrity of the CD1b hydrophobic channels is maintained by association with endogenous phosphatidylcholine and a long endogenous spacer (EnSpacer). Together, these two lipids stabilize the CD1b groove (15).

The crystal structure of SGL12 in complex with CD1b [Protein Data Bank (PDB) entry 3T8X] (18) shows the sulfotrehalose head exposed on the binding groove, while the EnSpacer is fully embedded inside the CD1b internal channels. In this structure, the F’ channel is closed due to a conformational rearrangement involving several binding groove residues, which prevents the EnSpacer from sliding outward (19). This, however, may not be the case for the natural Mtb sulfoglycolipid. While the synthetic analog SGL12 has a fixed-length lipid moiety, the Ac_2_SGL antigen shows heterogeneous lengths, with its most abundant variant having a 32 carbon hydroxyphthioceranoic acid containing eight branched methyl groups. In contrast, the corresponding chain in SGL12 has 24 carbons in total, with only four branched methyl groups (14).

In consequence, presentation of the native Ac_2_SGL antigen on CD1b most likely requires a repositioning of the EnSpacer.

The discovery of new specific ligands capable of specifically detecting Mtb-infected cells may contribute to the development of new diagnostic and therapeutic tools. On the other hand, the recognition of infected cells by ligands such as antibodies implies the recognition of Mtb-specific antigens presented on surface molecules such as HLA and CD1. In this sense, ligands recognizing HLA-Mtb-epitopes and CD1b-Mtb-lipids complexes have been reported (20–22), but the high polymorphism of the HLA molecules is a drawback for their use as universal markers for infected cells, so, antigens bound to the non-polymorphic CD1 molecules, offer an interesting alternative as universal markers of Mtb infected cells.

Using the phage display technology, our group obtained a single-chain T cell receptor (scTCR) construct, composed of the variable alpha and beta domains from the Z4B27 clone. This recombinant scTCR recognizes both the CD1b-Ac_2_SGL and CD1b-SGL12 complexes, showing a higher reactivity for the complex with the natural antigen. The phage-displayed scTCR was also able to recognize Mtb-infected cells from a TB patient (23), showing the potential to become a diagnostic tool. Interestingly, a very similar behavior was found for a Vk (variable kappa) domain antibody fragment (dAbκ11) selected from a phage display library using CD1b-transfected cells loaded with Ac_2_SGL (20).

Taken together, the experiments recapitulated here, both the functional studies, at the cellular level (using the Z4B27 clone, with cytokine-release assays) (13, 14, 24), and the binding studies at the molecular level (using the scTCR or dAbκ11 in ELISA studies) (20, 23), show a marked dependence between the acyl chain length and the TCR recognition of the CD1b-sulfoglycolipid complexes. On the other hand, and as pointed above, the crystal structure of the CD1b:SGL12 complex provides important clues on how a much larger acyl chain would push the EnSpacer, so much that it might not fit completely inside the CD1b channels. This, in turn, might directly affect the TCR binding event.

Here we put forward the hypothesis that the CD1b EnSpacer plays an important, direct role in the recognition of the CD1b-Ac_2_SGL complex by specific T cells, and specifically by the scTCR derived from the Z4B27 clone. To give support to this hypothesis we first performed additional binding assays, for both the scTCR and dAbκ11, against a small panel of synthetic Ac_2_SGL analogs having different acyl chains, and then constructed a computer model of the CD1b-Ac_2_SGL/EnSpacer complex, which was subsequently used for modeling of the interactions of this complex with the scTCR.

## Materials and Methods

### CD1b:Lipid Complexes

Ac_2_SGL, the synthetic analogs (SGL12, SL1, SL37, and SL38) and human sulfatide (hSulf), Avantis Polar Lipids (United States), were complexed in solution with recombinant human CD1b as described by Garcia-Alles et al. (18, 19).

### Ligands

M13 phage displaying scTCR and a light chain κ domain antibody (dAbκ11) recognizing CD1b-Ac_2_SGL were obtained as described by Camacho et al. (20, 23).

### Enzyme-Linked Immunosorbent Assay

A 96-well Maxisorp microplate (Nunc, United States) was coated (16 h/4°C) with: (a) the anti-CD1b mAb BCD1b3.1 (Sigma, United States) to capture CD1b complexes and free CD1b; (b) the anti-CD1e mAb CD1e20.6 to capture the recombinant human CD1e. Both mAbs were diluted in carbonate-bicarbonate pH 9.6 coating buffer (3 μg/mL). The plate was then blocked with 3% PBS-skim milk (1 h/RT). Two washes (PBS-tween-20, 0.05%) were performed, and the complexes and free CD1b were added in PBS pH7.4 (1 μg/mL). After three washes (PBS-tween20, 0.05%), scTCR and dAbκ11, diluted in 3% PBS-skim milk (10^8^ phages) were added and the plate was incubated for 1 h at RT. Subsequently, three washes were performed, and HRP/Anti-M13 monoclonal conjugate (GE, Healthcare, Life Sciences; diluted 1:5000 in 3% PBS-skim milk) was added. 1 h later, the plate was washed as above, TMB liquid substrate (Sigma, United States) was added, followed by incubation (10 min/RT). The reaction was stopped with 1 M sulfuric acid (Merck, Germany). Absorbance values (450 nm) were measured in a microplate reader (BioRad, United States). Each sample was studied in triplicate, and the experiment was repeated three times.

### Statistical Analysis

Analysis of variance (ANOVA) was performed to compare absorbance values, and the Tukey multiple comparisons test was then performed to determine significant differences in the recognition of the different CD1b-lipid complexes by scTCR and dAbκ11. Statistical analyses were performed using GraphPad Prism version 4.0 (San Diego, California, United States).

### Construction of the CD1b:Ac_2_SGL:EnSpacer Complex

The program VMD v1.9.3 (25) was used for most modeling procedures. The crystal structure of the CD1b:SGL12 complex (19) (PDB entry 3T8X) was used as main template both for the protein and the lipid ligands. Most of the CD1b structure was in 3T8X was kept as is, with the exception of the backbone and side chain conformations of a group of residues surrounding the F’ channel way out, which were copied from 1GZP (17). The program Avogadro v1.2 (26), was employed to modify the SGL12 structure into Ac_2_SGL and to complete the modeled EnSpacer out of a fragment of the crystal spacer of 3T8X. Torsion angles in the Ac_2_SGL acyl chain and in the EnSpacer were manipulated using the Molefacture plugin in VMD.

### Construction of the CD1b:Ac_2_SGL:EnSpacer Complex With the scTCR

The starting model for the scTCR was constructed with the Swiss Model Server (27), based on our manually curated alignment of the target and template sequences. The linker joining the alpha and beta chains was not modeled. The TCR of PDB entry 4EN3 was chosen among several possible templates because of the high percent of amino acid identity shown for both the alpha (88.7%) and beta (48.6%) variable domains. Then the full model of the CD1b:Ac_2_SGL:EnSpacer:scTCR complex was assembled using the CD1b-TCR complex in 5WKI as template. The obtained models of CD1b:Ac_2_SGL:EnSpacer and scTCR were superimposed on their protein counterparts in 5WKI using as reference atoms the alpha carbons of selected sequence segments corresponding to structurally conserved helices and/or beta sheet strands. The program CHARMM v43a1 (28) was used to run a one nanosecond (1 ns) molecular dynamics simulation at 400 K in vacuum only for the TCR, keeping fixed most of its structure and allowing to move selected CDR residues, including most of CDR3α and CDR3β. A representative conformation, sterically compatible with the lipid ligands, was then selected out from the collected 1,000 frames. The final model was obtained from a molecular dynamics simulation of the whole complex in a water box, aimed to refining the crude starting model and explore the intermolecular interactions.

## Results

### Binding Data Shows a Decrease in Reactivity for Synthetic Analogs With Shorter Acyl Chains

**FIGURE 1:**
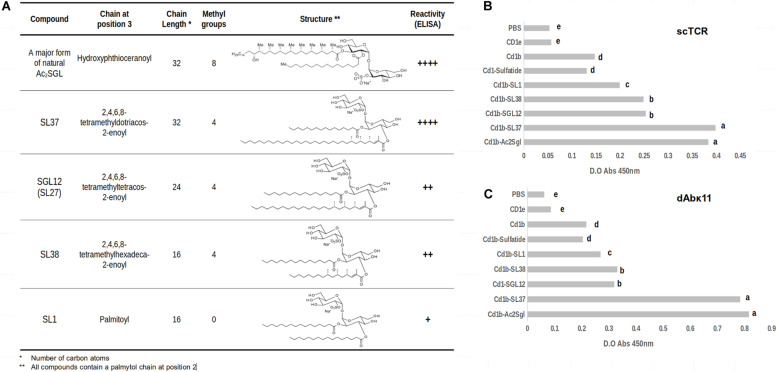
Differential recognition of CD1b:Mtb lipid complexes by phage-displayed ligands. **(A)** Structures and characteristics of *M. tuberculosis* sulfoglycolipid (Ac_2_SGL) and synthetic sulfoglycolipid analogs. **(B,C)** Recognition of CD1b:Mtb Ac_2_SGL and its synthetic analogs complexes by the phage-displayed scTCR and dAbvk11 ligands, respectively, as measured by ELISA. The data represent the means of the absorbance values of three replicates +SD. Lowercase letters represent statistically significant differences.

To further assess the influence of different acyl chain lengths in recognition of the sulfotrehalose head by the scTCR, we used a panel of molecules consisting of the natural antigen Ac_2_SGL and four synthetic analogs (Figure 1A). Their code names and the structures of their acyl chains are shown in Figure 1A. In three of the synthetic analogs (SL37, SGL12, and SL38), the acyl chain is tetramethylated, while in the SL1 analog it is completely linear.

Figure 1B shows the reactivity of the scTCR with these analogs presented on a recombinant CD1b receptor, as measured by enzyme-linked immunosorbent assay (ELISA). The natural antigen Ac_2_SGL and the SL37 analog were recognized by the scTCR with the same strength. They both have acyl chains with the same length (32 carbons) but differing in the number of methyl branches (eight vs. four). Then we observed a marked decrease in reactivity for the SGL12 analog, whose acyl chain is tetramethylated as that of SL37 but is much shorter (24 carbons). Noteworthy, the crystal structure of SGL12 in complex with CD1b shows that three of the four methyl branches are exposed to the solvent on the CD1b binding groove, while the fourth branch is placed at the very entrance of the A’ channel. Thus, taken together, these results suggest that the additional four methyl branches found in Ac_2_SGL (namely in its most abundant variant), are inserted deeply in the A’ channel and therefore have little or no influence in scTCR recognition. A further decrease in length of the acyl chain (down to 16 carbons, in SL38) but keeping the four methyl branches, did not have an additional effect in scTCR reactivity since the recognition levels for SGL12 and SL38 are practically the same. Finally, a further slight decrease in reactivity was observed for SL1, having a 16-carbon acyl chain, but completely linear.

A key point implicitly included in the above analyses is that the orientation of the sulfotrehalose head on the CD1b groove must be extremely similar for all these sulfoglycolipids. The reason for this is that the scTCR binds to all these antigens while docked to CD1b, which it does in a well conserved orientation, as discussed further below. In other words, in all these binding events the scTCR pocket that takes in the sulfotrehalose head is placed in the same conserved position on the CD1b groove. Curiously, the reactivity of the dAbκ11 antibody domain, which we tested here for binding to the same CD1b-bound synthetic analogs, followed the same pattern observed for the scTCR. However, the native antigen and SL37 were recognized with higher reactivity as compared to the rest of the analogs (Figure 1C).

These results led us to hypothesize that the cause behind the observed differences in recognition by the scTCR (and most likely also by dAbκ11) is a displacement of the EnSpacer due to the long fatty acid chain in Ac_2_SGL and SL37, which forces the EnSpacer to protrude above the CD1b groove. The lipid presented this way, may interact with the TCR and, possibly, modulate its binding reactivity and the elicited immune response.

### Analysis of EnSpacer Arrangement in CD1b Crystal Structures

We looked for CD1b crystal structures to investigate if any of them contained a protruding EnSpacer. A search in the PDB yielded 13 entries presenting different antigens (PDB codes: 1GZP, 1GZQ, 1UQS, 2H26, 3T8X, 5L2J, 5L2K, 5WKE, 5WKG, 5WKI, 5WL1, 6CUG, and 6D64); three of these entries contain CD1b-antigen complexes with T-cell receptors. The relatively small number of structures was tractable for individual visual inspection. None of the structures displayed the EnSpacer protruding above the level of the groove borders, namely above the level of the Tyr151 side chain (18). Moreover, several of these complexes showed a closed F’ channel, holding the spacer completely buried.

Noteworthy, in one of the structures – 1UQS, CD1b in complex with glucose monomycolate (GMM) – the EnSpacer is absent because the long meromycolate chain of GMM spans all the way from the A’ to the F’ channel. In contrast, in the other 12 structures, the fatty acid chains are accommodated in the A’ channel, with approximately half of them reaching up to the turn connecting the A’ and T’ channels. Compared to these farther-reaching fatty acid chains, the end of the SGL12 acyl chain stays behind by about three carbon atoms. On the other hand, the EnSpacers modeled on the electronic density maps in these structures show different lengths, most of them between 34–40 carbon-atoms, except for the short lipids (≤26 carbons) seen in 5L2J, 5L2K, and 5WKI.

In summary, except for the large GMM antigen, EnSpacers of different lengths are lined up with the acyl chains of the other CD1 ligands along the T’ and F’ channels, without protruding above the CD1 binding groove. None of these acyl chains, however, is as long as the hydroxyphthioceranoic chain of Ac_2_SGL.

### Model of the CD1b:Ac_2_SGL Complex With a Protruding EnSpacer

The fatty acid chain of the most abundant Ac_2_SGL variant is 32-carbon long, which exceeds the length of the corresponding chain in SGL12 by eight carbons. If the sulfotrehalose head in both molecules adopts the same position on the CD1b binding groove, then the acyl chain of Ac_2_SGL most likely enters the T’ channel, displacing the EnSpacer toward the F’ channel exit. To assess this effect, we constructed a homology model of the CD1b:Ac_2_SGL complex, based mostly on the crystal structure of the CD1b:SGL12 complex (3T8X), with a few residues at the F’ channel exit being modeled based on the crystal structure of the CD1b:GM2 complex [1GZP (17)].

**FIGURE 2:**
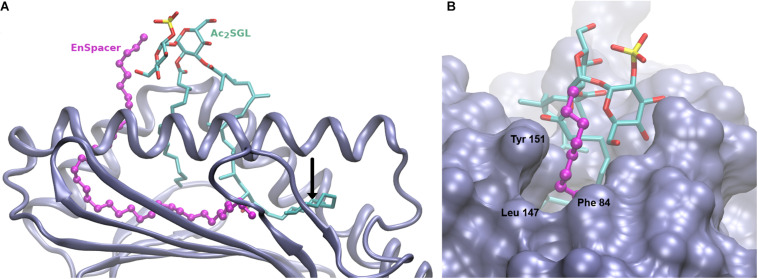
Model of the CD1b:Ac_2_SGL:lipid complex. **(A)** Overall view showing the Cd1b domains α1 and α2 (in ice blue cartoon), the Ac_2_SGL antigen (in sticks, colored by atom type), and the 38-carbon endogenous lipid (in balls and sticks, magenta). The tail of the acyl chain of Ac2SGL makes a turn and enters into the T’ channel, displacing the endogenous lipid. The vertical black arrow indicates the position that would be occupied by the last carbon atom of the 24-carbon long acyl chain of SGL2. The spacer in the CD1b:SGL12 crystal complex would then be positioned right after it, filling up the space occupied by the remaining Ac2SGL tail. **(B)** Zoom on the F’ channel way out, showing the protruding extreme of the endogenous lipid above the binding groove. CD1b in molecular surface representation, Ac2SGL and the lipid as in panel **A**.

Figure 2 shows the resulting model. As indicated above, the sulfotrehalose head of Ac_2_SGL was kept in the same conformation and relative position as in the CD1b:SGL12 complex, therefore its interactions with the CD1b receptor are the same observed in the template structure. The hydroxyphthioceranoic chain of Ac_2_SGL was built, as much as possible, on the SGL12 acyl chain, while the exceeding eight linear carbons were copied on the carbon chain of the crystal spacer (Figure 2A). Then we modeled a 40-carbon long EnSpacer, most of which (30 carbons) was built on the crystal spacer in 3T8X. The remaining 10 carbons were added following the stretched conformation observed in 1GZP as well as in several other CD1b structures. As expected, a six-carbon segment of the EnSpacer protrudes above the groove borders (Figure 2B). This segment was modeled bended over the closest trehalose ring. Still, because of its high flexibility, it may easily adopt other conformations, for example, bending over the hydrophobic patch formed by Phe84 and Leu147 at the F’ channel way out. In any case, even for slightly shorter spacers, this extreme of the EnSpacer becomes exposed to possible interactions with T cell receptors.

### A Model of the Complex of CD1b:Ac_2_SGL:EnSpacer With the scTCR Shows Direct Participation of the EnSpacer in the Interaction

Having the model of the CD1b:Ac_2_SGL:EnSpacer complex, we undertook the construction of a model of this molecular system in complex with the variable domains of the Ac_2_SGL-specific scTCR derived from the Z4B27 clone. The three crystal structures of CD1b-antigen-TCR complexes deposited so far in the PDB 5L2K (29); 5WKI (30); and 6CUG (31) show that these TCRs are anchored on CD1b in a conserved orientation (31), which provides a reliable ground for homology modeling of the scTCR complex with CD1b:Ac_2_SGL.

First, a model of the scTCR variable domains was constructed using the SwissModel server, after a careful selection of the TCR template (4EN3) and supervised alignment of its alpha and beta domain sequences with the corresponding scTCR sequences, aiming to produce an accurate model. Then, the models of the CD1b:Ac_2_SGL:EnSpacer complex and the scTCR were superimposed on the corresponding CD1b and TCR molecules in PDB entry 5WKI, chosen as a template to assemble the full molecular model.

**FIGURE 3:**
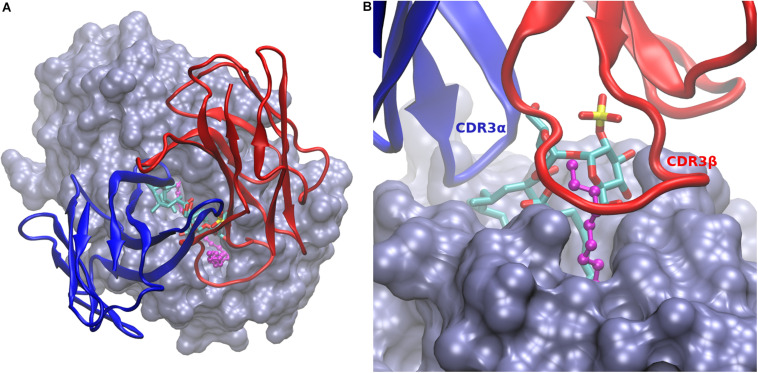
Model of CD1b:Ac_2_SGL:lipid in complex with the scTCR. **(A)** Overall view of the scTCR docked on top of the CD1b:Ac_2_SGL:lipid complex. CD1b in ice blue molecular surface, Ac2SGL in sticks (colored by atom type), and the endogenous lipid in balls and sticks (magenta). The alpha (blue) and beta (red) TCR chains are represented in cartoon. **(B)** Zoom on the binding interactions from the TCR beta chain side. CDR3β (labeled in red) embraces the lipid together with the trehalose head, while CDR3α (labeled in blue) interacts with the sulfoglycolipid from the opposite side.

The obtained model needed some additional adjustments, in particular for the long CDRs 3 of both the alpha and beta variable regions, which play a principal role in the interaction with the presented antigen (31). While most of the scTCR structure, including CDRs 1 and 2 of both chains, could be modeled with confidence, the long and flexible CDRs 3 was built by the automated server in one its many possible conformations, in which these loops bump onto the trehalose head and the protruding EnSpacer. To explore the conformational space of both CDR3 loops for suitable binding geometries, we conducted a short (1 ns) molecular dynamics simulations in vacuum, in which the two CDRs 3 and a few amino acids at the tips of the other four CDRs were allowed to move, while the rest of the TCR structure remained fixed. From the collected ensemble of conformations (1,000 frames), we selected a representative geometry where both CDRs 3 embraced the trehalose and lipid heads. A final model was then obtained from a molecular dynamic simulation of the whole complex in a water box (Figure 3).

In the model, the sulfotrehalose head of Ac_2_SGL fits well in a cavity formed mainly by CDR3α and CRD3β, with contributions also from CDR1β and CDR2β. The long and flexible CDR3β embraces the protruding segment of the EnSpacer together with a trehalose ring and its sulfate group and extends further over the CD1b surface (Figure 2B). On the opposite side, CDR3α covers the other trehalose ring and also the exposed part of the acyl chain containing the first three methyl branches. In general, the model provides a rational structural framework to explain the scTCR specificity.

## Discussion

The relevance of chain length and methylation of the fatty acids in Ac_2_SGL synthetic analogs, regarding their capability to stimulate specific T-cell clones, has been demonstrated in several studies – longer acyl chains are more antigenic (14, 24). In this work, we propose and give support to a plausible structural explanation for these experimental findings, whose cornerstone is the direct participation of the EnSpacer, forced to protrude on the CD1b groove, in the interactions with T-cell receptors.

We selected a small panel of molecules consisting of the natural Mtb antigen Ac_2_SGL and four synthetic analogs – three of them having tetramethylated acyl chains of different lengths (32, 24, and 16 carbons) and one analog having a 16-carbon linear chain – to further characterize the recognition capabilities of the recombinant scTCR derived from the CD1b-Ac_2_SGL specific T cell clone Z4B27, in particular, its dependence on acyl chain length. In parallel, we also tested the antibody Vk domain dAbκ11. Our molecular panel included the SGL12 analog (C24, tetramethylated), previously tested for binding of both the scTCR and dAbκ11.

As shown by Gau et al. (24), the length of tetramethylated fatty acyl chains is crucial for efficient T-cell activation – the longer the chain, the more stimulatory the analog. In fact, their synthetic analog 21c (32-carbon long) is almost identical to the SL37 analog used here, except for the saturation of the fatty acid, which was found to have no effect in the antigenic properties of the tested compounds (24). Our molecular binding assays showed a similar dependence on acyl chain length and methylation, found in previous cellular assays (14, 24).

By contrasting these binding and functional cellular-assays results with the available structural data, we inferred that a common EnSpacer (36–40 carbon long), when accommodated together with a long acyl chain (e.g., 32 carbon long), has to protrude above the F’ channel way out. To support this hypothesis, we constructed a structural model of CD1b in complex with Ac_2_SGL together with an average-length EnSpacer. A key result to back up our model was the observed equal binding of the scTCR and dAbκ11 to Ac_2_SGL and SL37, which differ in their number of methyl branches but have the same acyl chain length. We interpreted this as a strong evidence that the trehalose units of both molecules are exposed in the same geometry on the CD1b binding groove. On the other hand, the acyl chains of both SL37 and SGL12 are tetramethylated, so they should accommodate themselves in practically the same way at the entrance of the A’ channel.

Based on these experimental facts and arguments, we concluded that the sulfotrehalose heads of Ac_2_SGL and SGL12 must be displayed on CD1b in the same spatial configuration and, therefore, the crystal structure of CD1b in complex with SGL12 (18) became an ideal template for homology modeling of the complex with Ac_2_SGL. The model of the CD1b:Ac_2_SGL:EnSpacer complex was built for the most abundant Ac_2_SGL variant and for an EnSpacer of average length (38 carbons). Still, according to the model, any slightly shorter or longer EnSpacer (within a 36–40 carbon range), would have a carbon stretch protruding on the CD1b groove and exposed to T-cell receptors, together with the sulfoglycolipid.

Currently, no structural data is available on possible TCR interactions involving the EnSpacer, since in the three crystal structures of CD1b-antigen-TCR complexes deposited so far in the Protein Data Bank (29–31) the lipid is completely buried in the CD1b molecule. However, an inspection of these structures revealed that in all of them the CDR3β covers the F’ channel exit, being in contact with CD1b amino acids at the closed end of the channel. Therefore, a protruding EnSpacer would most likely be in contact with CDR3β.

Our model of the whole CD1b:Ac_2_SGL:EnSpacer:scTCR complex illustrates how such interactions may occur. The constructed model has strong as well as weak points. On the strong side: (a) the CD1b:Ac_2_SGL component has a solid ground in the crystal structure of the CD1b:SGL12 complex; (b) most of the scTCR structure, specifically its framework and CDRs 1 and 2 of both the alpha and beta chains, could be reliably modeled based on highly similar in sequence TCR structures; and (c) the conserved TCR orientation observed in the CD1b-TCR crystal complexes (31) allowed a safe initial docking of the scTCR model onto the CD1b-antigen complex. On the other side, the weak points of the model are mainly two: (a) the conformations of the highly flexible backbones of the two long CDRs 3 (alpha and beta) and their side chains cannot be modeled with confidence; and (b) the protruding segment of the EnSpacer is highly flexible, making difficult to predict its right complexed conformation. But in spite of the uncertainties, inherent to most computer models of protein-protein complexes (32) the model of the CD1b:Ac_2_SGL:EnSpacer:scTCR complex shows that even a short protruding segment of the EnSpacer would interact with the CDR3β of the scTCR and, most likely, with other Ac_2_SGL-specific T-cell receptors as well.

## Conclusion

In this study, we describe the interactions of Ac_2_SGL with CD1b and provide support to the hypothesis that its long acyl chain forces the direct engagement of the EnSpacer in TCR recognition. These results contribute to understanding the mechanisms of lipid presentation by CD1b molecules and their interactions with T-cell receptors and other specific ligands, which will help to develop specific tools targeting Mtb infected cells for therapeutic and diagnostic applications.

## Data Availability Statement

The original contributions presented in the study are included in the article/Supplementary material, further inquiries can be directed to the corresponding author/s.

## Author Contributions

All authors have read and approved the manuscript and contributed significantly to this work.

## Conflict of Interest

The authors declare that the research was conducted in the absence of any commercial or financial relationships that could be construed as a potential conflict of interest.

## Abbreviations

Ac_2_SGL: 2-palmitoyl or 2-stearoyl-3-hydroxyphthioceranoyl-2 ′ -sulfate- α - α ’-d-trehalose
dAb κ 11: domain antibody
EnSpacer: endogenous spacer that stabilizes the CD1b groove
Mtb: *Mycobacterium tuberculosis*
scTCR: single chain TCR
TB: Tuberculosis
